# Chronic hepatitis B and perioperative chemotherapy efficacy in colorectal liver metastases: an exploratory analysis of clinical and mechanistic evidence

**DOI:** 10.3389/fimmu.2026.1914602

**Published:** 2026-09-04

**Authors:** Chun-Liang Cheng, Shan-You Tong, Shao-Bo Mo, San-Jun Cai, Jun-Jie Peng, Xiang Hu

**Affiliations:** 1 Department of Colorectal Surgery, Fudan University Shanghai Cancer Center, Shanghai, China; 2 Department of Oncology, Shanghai Medical College, Fudan University, Shanghai, China

**Keywords:** adjuvant chemotherapy, colorectal liver metastases, liver surgery, perioperative chemotherapy, TSPAN8

## Abstract

**Background:**

The optimal choice of chemotherapy regimen for resectable colorectal liver metastases (CRLM) is still undetermined. The study aimed to estimate the impact of perioperative or adjuvant chemotherapy on disease-free survival (DFS), and explore the underlying mechanisms which influence chemotherapy efficacy.

**Methods:**

Eight hundred twenty-two patients undergoing curative resection of CRLM were retrospectively collected from May 2018 to December 2023. Treatment effects between perioperative and adjuvant chemotherapy were compared in full subgroup analyses by Kaplan–Meier and Cox proportional hazards methods. Single-cell RNA sequencing and tumor derived organoids were used to explore the molecular mechanisms.

**Results:**

After propensity score matching, 630 patients were enrolled with ratio 1:1 in adjuvant and perioperative chemotherapy group. The median DFS in adjuvant and perioperative group was comparable with 28 and 32.5 months, respectively (HR = 0.99, *P* = 0.945). Perioperative chemotherapy was associated with improved DFS (25 vs. 13 months, *P* = 0.039) in patients with a clinical risk score (CRS) of 5. Notably, within the high-risk CRS 4-5 population receiving perioperative chemotherapy, patients with chronic hepatitis B (CHB) had significantly worse DFS (HR = 4.31, 95% CI 1.93–9.64; *P* < 0.001). Single-cell analyses revealed TSPAN8^+^ stem-like epithelial cells were enriched in sample with CHB. TSPAN8 knockdown sensitized colorectal cancer cells to 5-fluorouracil, and anti-TSPAN8 antibody showed synergistic efficacy with 5-fluorouracil in patient-derived liver metastasis organoids. In addition, the enhanced SPP1-CD44 signaling between TSPAN8^+^ epithelial cells and SPP1^+^ macrophages also contributed to 5-fluorouracil resistance.

**Conclusions:**

Higher CRS identify a subgroup of CRLM patients who may derive greater benefit from perioperative chemotherapy. HBV-related serological profiles is a potential predictor of chemotherapy resistance. The exploratory findings warrants further validation.

## Introduction

Surgical resection is the cornerstone of curative treatment for colorectal liver metastases (CRLM), which achieves 5-year and 10-year overall survival (OS) rates of approximately 40% and 35%, respectively (1, 2). Nevertheless, the risk of postoperative recurrence remains substantial, up to 60% patients experience relapse within the first two years (1). Thus, it is necessary to integrate chemotherapy into standard treatment paradigms to improve disease-free survival (DFS) (3, 4). Currently, guidelines from the European Society of Medical Oncology and other authoritative institutions recommend perioperative or adjuvant chemotherapy in combination with curative resection for CRLM (3, 5, 6).

Perioperative chemotherapy constitutes a sequential therapeutic strategy that includes neoadjuvant chemotherapy and adjuvant chemotherapy postoperatively. Compared with adjuvant chemotherapy alone, perioperative chemotherapy is generally considered the most feasible approach especially in patients with uncertain oncological prognosis or high postoperative relapse risk (7). Perioperative chemotherapy has several theoretical advantages, including early control of micrometastatic disease, *in vivo* evaluation of tumor chemosensitivity, and potential downstaging of liver metastases. However, perioperative chemotherapy also had inherent risks: chemotherapy-related liver toxicity, and disease progression during neoadjuvant treatment which may complicate or even preclude curative resection (8, 9).

The clinical risk score (CRS) is widely adopted to stratify recurrence risk in patients with CRLM, in which CRS of 0–1 are classified as low-risk, while 3–5 represent high-risk (10). Current guidelines recommend perioperative chemotherapy for high-risk patients with CRS scores of CRS 3–5, yet the optimal risk threshold for initiating perioperative chemotherapy remains undefined. Direct comparative evidence between perioperative and adjuvant chemotherapy is limited, as several randomized trials were prematurely terminated due to poor accrual (11–13). Furthermore, prior observational studies were constrained by heterogeneous patient selection and insufficient adjustment for biological covariates (14, 15). Consequently, no consensus has been reached regarding the optimal therapeutic regimen for resectable CRLM.

In this study, we aimed to identify the optimal population that benefit from perioperative chemotherapy based on the CRS system. Considering the high prevalence of hepatitis B virus (HBV) infection in China and its potential impact on the hepatic microenvironment, we specifically included HBV infection status as a critical stratifying factor. Additionally, we performed exploratory single-cell transcriptomic profiling to investigate how HBV infection influences treatment response.

## Materials and methods

### Study population

Patients with colorectal liver metastases were collected. All patients had received radical primary and liver lesions resection or ablation from May 2018 to December 2023. Patients with extrahepatic metastasis, R1 resection, or hepatic arterial infusion chemotherapy previously used, immunotherapy or target therapy previously used, and those receiving fewer than four cycles of chemotherapy were excluded. Patients with microsatellite instability-high were also excluded due to the priority of immunotherapy. Eligible patients were divided into two groups: the adjuvant chemotherapy group and the perioperative chemotherapy group. Chemotherapy was administered according to standard first-line regimens for CRLM, including oxaliplatin-based, irinotecan-based, or combination oxaliplatin and irinotecan regimens.

Patients’ baseline demographic information, characteristics of primary colorectal tumors and liver metastases were retrospectively retrieved. HBV associated serological markers were also collected at the time of surgical admission, including hepatitis B surface antigen (HBsAg), hepatitis B surface antibody (HBsAb), hepatitis B e antigen (HBeAg), hepatitis B e antibody (HBeAb), and hepatitis B core antibody (HBcAb). In this study, chronic hepatitis B (CHB) was defined as persistent HBsAg positivity for more than six months, and occult hepatitis B infection (OHB) was defined as detectable HBcAb or HBeAb in the absence of HBsAg. Notably, all patients in the cohort tested negative for HBeAg, indicating that none were in an acute phase of HBV infection prior to surgery. Therefore, aberrant HBV-related serology was defined as CHB or OHB, whereas normal HBV-related serology was defined as negative for HBsAg, HBeAg, HBcAb, and HBeAb.

The study was conducted in accordance with the Declaration of Helsinki and approved by the Ethics Committee of Fudan University Shanghai Cancer Center. The study was registered at www.researchregistry.com (Registration Number: researchregistry11008).

### Follow-up protocol

In our center, hepatic resection was performed according to parenchyma-preserving surgery with preoperative MRI and intraoperative ultrasound-guided navigation (16). Hepatic resection was scheduled four weeks after the neoadjuvant chemotherapy. The number and size of metastatic lesions were evaluated preoperatively by MRI and intraoperative ultrasound.

Follow-up was mainly performed through outpatient clinic visits every 3–6 months within the first 5 years post-operatively, and once per year thereafter. Physical examination, tumor biomarkers, chest and abdominal CT and MRI were performed as well as colonoscopy once per year after the surgery. The first follow-up was initiated at liver resection, and ended at the evidence of tumor relapse. Patient surveillance was closed at the end of February 2024. The long-term prognostic outcomes during follow-up were retrieved and downloaded from a routinely maintained database at our institution.

### Propensity score matching

Propensity score matching (PSM) was performed to reduce confounding bias of baseline characteristics. The propensity score was defined as the conditional probability of a patient being assigned to perioperative chemotherapy versus adjuvant chemotherapy, given observed preoperative covariates. The propensity score model included all clinically relevant preoperative variables that may influence treatment selection (17). And propensity scores were estimated using a logistic regression model with chemotherapy strategy as the dependent variable and baseline prognostic factors as covariates. A 1:1 nearest-neighbor matching was performed with a 0.2 standard deviation caliper on the logit of the propensity score (18). To assess the balance of covariates after PSM, we calculated standardized mean differences (SMD) for all baseline variables, with values below 0.1 indicating good balance.

### Single-cell RNA sequencing analysis

Single-cell analysis was performed to explore the mechanism underlying the blunted benefit of perioperative chemotherapy in HBV-infected CRLM patients. Publicly available single-cell RNA sequencing data (GSE235057) which comprised with 9 CRLM samples were retrieved from the Gene Expression Omnibus (https://www.ncbi.nlm.nih.gov/). The clinical information including HBV status was extracted from the supplementary tables of the original publication (19).

Seurat package was used for data processing. Genes expressed in ≥3 cells and cells with ≥200 detected genes were retained. Cells with >20% mitochondrial content, <1000 or >50,000 RNA counts, or >5000 detected genes were excluded. Data were normalized, scaled, and batch-corrected using Harmony. Principal component analysis was performed on highly variable genes, followed by graph-based clustering (Louvain algorithm) and UMAP visualization. Eight cell types were annotated based on canonical marker genes.

### Differential expression analysis

Differentially expressed genes (DEGs) were identified using FindAllMarkers with thresholds of |log2FC| > 0.7 and adjusted p-value < 0.05.

### Biological activity inference

Gene Set Variation Analysis (GSVA) was applied to estimate the functional heterogeneity of cancer cells. We referenced the CancerSEA database, a specialized resource that comprehensively decodes distinct functional states of cancer cells. In addition, we manually compiled 26 stemness-related gene sets to construct comprehensive reference. UCell package was used to estimate these single-cell signatures. And the complete list of gene sets is provided in **Supplementary material**.

### Observed-to-expected ratio

The Ro/e method was used to assess cell type enrichment. The Ro/e value was calculated by constructing a contingency table of cell type annotations and sample groups to compare the observed cell counts with the expected counts under the null hypothesis of random distribution.

### Scissor algorithm

The scissor algorithm was applied to integrate bulk transcriptomic data and paired survival information to single-cell RNA sequencing data. Cells with positive coefficients were defined as scissor^+^ cells, representing a high-risk cell subpopulation associated with inferior survival. Scissor^-^ cells were defined as a low-risk cell subpopulation correlated with favorable prognosis and chemotherapy sensitivity.

### Trajectory analysis

Pseudotime trajectory analysis was performed by Monocle package based on cluster-specific marker genes. Cell differentiation trajectories were inferred and visualized using DDR-Tree with default parameters.

### Cell-cell interaction analysis

Cell-cell communication between epithelial cells and macrophages was analyzed by CellChat package. Significant ligand-receptor pairs (*p* < 0.05) were selected for downstream visualization. Interaction networks were plotted, and communication probabilities were displayed as bubble plots.

### HCT-8 resistant cell line

To validate the role of TSPAN8 in 5-fluorouracil resistance, we utilized the publicly available RNA-seq dataset GSE81005, which contains global gene expression profiles of parental HCT-8 cells and their 5-fluorouracil-resistant subline (HCT-8/5-FU). The resistant cell line was established by continuous exposure to increasing concentrations of 5-fluorouracil. Raw expression data were downloaded from the Gene Expression Omnibus. Differentially expressed genes were identified, and TSPAN8 expression levels were compared between the parental and resistant cells.

### Cell culture

Human colorectal cancer cell line SW620 and HT29 was purchased from the American Type Culture Collection. The mycoplasma contamination was excluded by mycoplasma detection kit (Servicebio, G1282). All cells were cultured in high-glucose DMEM (Gibco, 11965118) supplemented with 10% fetal bovine serum (Gibco, A5670701) and 1% penicillin-streptomycin (Servicebio, G4003) at 37°C in a humidified incubator with 5% CO_2_.

### Gene knockdown

Small interfering RNA (siRNA) targeting TSPAN8 and CD44 was synthesized for gene knockdown. SW620 cells were seeded into 6-well plates 24 hours prior to transfection, then transfected with siRNA by using Lipofectamine RNAimax (ThermoFisher, 13778030). The medium was replaced after 6 -hours transfection. Total RNA and protein were extracted 48, 72 hours post-transfection, respectively. The siRNA sequences are provided in the **Supplementary Material**.

### Real-time PCR

Total RNA was extracted from cells by the SteadyPure Universal RNA Extraction Kit according to the manufacturer’s instructions. RNA was then reverse-transcribed into cDNA using a one-step cDNA synthesis premix kit. Quantitative real-time PCR was performed on a 7300Plus Real-Time PCR System using SYBR Green Fast qPCR Mix. GAPDH was used as the internal control, and each experiment was performed in triplicate. Primer sequences and the calculation of relative gene expression by using the 2^-^ΔΔCt method are provided in the **Supplementary Material**.

### Western blot analysis

Total proteins were extracted from cells, separated by sodium dodecyl sulfate-polyacrylamide gel electrophoresis, and transferred onto nitrocellulose membranes. The membranes were blocked with 5% non-fat milk, and incubated with primary antibodies against TSPAN8 (Cell Signalling Technology, 65237T) and β-actin (proteintech, 66009-1-Ig) overnight at 4°C. After the incubation with horseradish peroxidase conjugated secondary antibodies, protein bands were visualized using an enhanced chemiluminescence detection system.

### CCK-8 assay

Colorectal cancer cells were seeded in 96-well plates at a density of 1,000 cells per well and cultured overnight to allow for cell adhesion. Then, cells were treated with 5-fluorouracil (Selleck, S1209) or anti-TSPAN8 antibody (MedChemExpress, HY-P990536). Cell viability was assessed at 72h post-treatment by CCK-8 assay. 10% CCK-8 reagent (Beyotime, C0039) was added to each well, incubated at 37°C in the dark for 1h, and the absorbance at 450nm was measured.

### Colony formation assay

Cells were seeded in 6-well plates at 1,000 cells per well. After 3-days attachment, cells were treated with 5-fluorouracil, anti-TSPAN8 antibody, or their combination. The medium was replaced every 7 days, and cultures were maintained for 14 days until visible colonies formed. Colonies were fixed with 4% paraformaldehyde, stained with 0.5% crystal violet, photographed, and counted.

### Organoid validation

Liver metastasis organoids were established as previously described (20). Fresh tumor tissues were obtained from surgically resected liver metastases with written informed consent. Tumor samples were mechanically and enzymatically dissociated into small fragments, embedded in Matrigel, and cultured in a colorectal cancer-specific organoid medium at 37°C with 5% CO_2_. The medium composition is detailed in the **Supplementary Material**.

For drug sensitivity testing, organoids were harvested, dissociated into small-cluster suspensions, and seeded in 96-well plates with Matrigel. After 24 hours of recovery, organoids were treated with 5-fluorouracil, anti-TSPAN8 antibody, or their combination in fresh medium. Bright-field images were captured on days 0, 3, and 7 to monitor organoid morphology.

### SPP1-CD44 interaction

SW620 cells were seeded in 96-well plates at 1,000 cells per well. After attachment, cells were treated with 5-fluorouracil alone or in combination with recombinant human SPP1 protein at 2 μg/mL or 5 μg/mL for 48 hours. To confirm that the effect of SPP1 is mediated through CD44, SW620 cells were transfected with siRNA targeting CD44. Following knockdown, cells were reseeded and treated with 5-fluorouracil alone or combined with recombinant 2 μg/mL SPP1 for 48 hours. Cell viability was measured by CCK-8 assay.

### Immunohistochemistry

IHC staining for TSPAN8 was performed on formalin-fixed, paraffin-embedded CRLM tissue sections by standard protocols. A semi-quantitative scoring system was applied: staining intensity was graded from 0 to 3 (negative to strong), and positive cell proportion was scored from 1 to 4 (0-25% to 76-100%). The final IHC score was obtained by multiplying these two scores.

### Statistical analysis

Continuous variables were tested for normality. Normal distributed data were presented as mean ± standard deviation and compared using the independent-samples t-test. Non-normally distributed data were expressed as median (interquartile range) and analyzed using the Mann–Whitney U test. Categorized data were descripted using contingency tables and assessed by chi-square test or Fisher exact test for demographic and tumor characteristics of patients in two groups. For bar graphs with continuous variables, Levene’s test was used to assess variance homogeneity. One-way ANOVA was performed to compare overall mean differences, followed by Dunnett’s test for multiple comparisons between each experimental group and the control group.

Survival curves were estimated by Kaplan–Meier method, and compared by log-rank test. For multiple subgroup comparisons, Bonferroni correction was applied, and corrected *P* values are reported. Multivariate Cox proportional hazards regression was performed to identify independent prognostic factors. The proportional hazards assumption was formally tested for all Cox models. The interaction tests were utilized for the subgroup analyses to evaluate whether the chemotherapy effect varied according to subgroups. Hazard ratios (HR) with 95% confidence intervals (CI) were calculated. A two-sided P value < 0.05 was considered statistically significant. Statistical analyses were performed by using the statistical software package SPSS (version 22.0, IBM SPSS Inc, Chicago, IL) and R software (version 4.4.1).

## Results

### Patients’ baseline characteristics

From May 2018 to December 2023, a total of 822 patients with resectable CRLM met the eligibility criteria of the study. Among them, 398 (32.2%) received adjuvant chemotherapy and 424 (67.8%) received perioperative chemotherapy. Compared with the adjuvant chemotherapy group, the perioperative group had a higher prevalence of T4 stage tumors, a younger age profile (≤60 years), and more extensive liver metastatic burden (**Supplementary Table 1**).

To achieve a well-balanced distribution of baseline, a propensity score was used to adjust those covariates. After 1:1 nearest-neighbor PSM, 315 patients were included in each group, and all covariates were well balanced between the two groups Notably, only 5/315 (1.6%) patients in the adjuvant chemotherapy group, and 1/315 (0.3%) patients in the perioperative chemotherapy group had clinically notable liver function abnormalities prior to the first cycle of chemotherapy. No patient in either group had imaging evidence of clinically significant liver cirrhosis or portal hypertension (**Table 1**).

**Table 1:** Baseline characteristics of the study population after propensity score matching.

Variables	Category	Adjuvant chemotherapy (N = 315)	Perioperative chemotherapy (N = 315)	SMD^*^	*P* value
Gender	Male	208 (66%)	198 (62.9%)	0.066	0.405
	Female	107 (34%)	117 (37.1%)		
Age	≥ 60 years	155 (49.2%)	167 (53.2%)	0.076	0.339
	< 60 years	160 (50.8%)	148 (46.8%)		
Histology	Adenocarcinoma	304 (96.5%)	311 (98.7%)	0.146	0.067
	mucinous Adenocarcinoma	11 (3.5%)	4 (1.3%)		
pT	T1	10 (3.2%)	8 (2.5%)	0.038	0.954
	T2	17 (5.4%)	19 (6.0%)		
	T3	254 (80.6%)	254 (80.6%)		
	T4	34 (10.8%)	34 (10.8%)		
pN	N0	93 (29.5%)	95 (30.2%)	0.014	0.978
	N1	136 (43.2%)	136 (43.2%)		
	N2	86 (27.3%)	84 (26.7%)		
Location	Right	98 (31.1%)	114 (36.2%)	0.145	0.110
	Left	211 (67.0%)	189 (60.0%)		
	Rectum	6 (1.9%)	12 (3.8%)		
Tumor size	≥ 3 cm	205 (65.1%)	197 (62.5%)	0.053	0.507
	< 3 cm	110 (34.9%)	118 (37.5%)		
Metastasis size	Mean ± SD	4.1 ± 1.9 cm	3.9 ± 2.0 cm	0.103	0.199
Metastasis number	≥ 2	291 (92.4%)	301 (95.6%)	0.133	0.094
	< 2	24 (7.6%)	14 (4.4%)		
CRS score	3	198 (62.9%)	178 (56.5%)	0.143	0.136
	4	84 (26.7%)	89 (28.3%)		
	5	33 (10.5%)	48 (15.2%)		
RAS/BRAF status	Mutation	81 (25.7%)	101 (32.1%)	0.140	0.079
	Wild-type	234 (74.3%)	214 (67.9%)		
HBV status	CHB	26 (8.3%)	25 (7.9%)	0.032	0.922
	OHB	155 (42.5%)	151 (44.1%)		
	Negative	134 (42.5%)	139 (44.1%)		
Liver function	Abnormal	5 (1.9%)	1 (0.3%)	0.131	0.100
	Normal	309 (98.1%)	314 (99.7%)		

SMD*, standardized mean difference. For multi-level categorical variables, the largest SMD among all levels is reported. SMD < 0.1 indicates good balance, and SMD < 0.2 is generally considered acceptable.

### Perioperative chemotherapy improved DFS in CRLM patients with CRS 5

**Figure 1:**
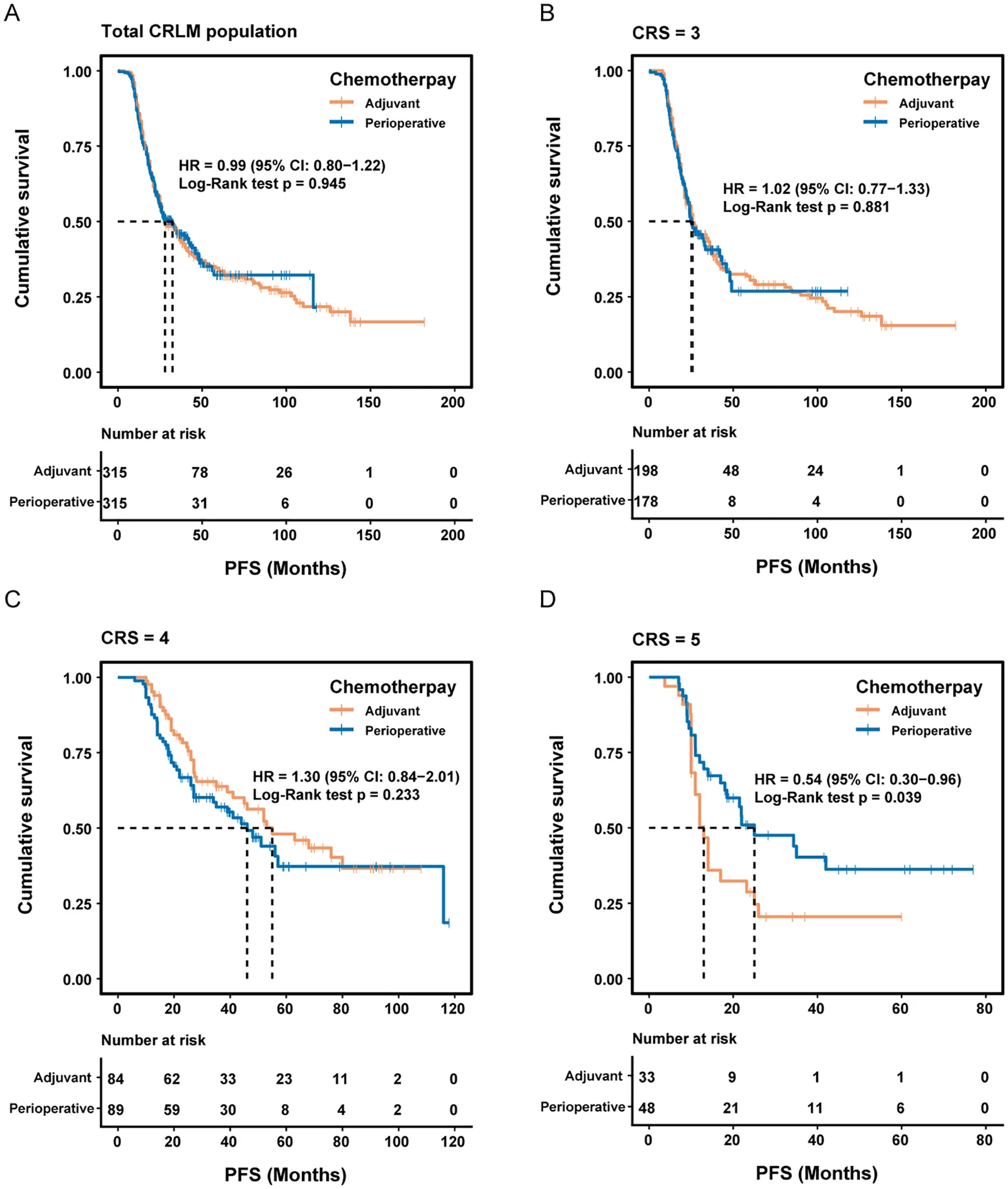
Kaplan-Meier estimates of DFS in the overall CRLM cohort and CRS-stratified subgroups. **(A)** DFS comparison between the adjuvant chemotherapy and perioperative chemotherapy groups in the overall cohort. **(B–D)** DFS comparisons between the two chemotherapy regimens stratified by CRS 3, 4, and 5, respectively. Risk tables indicate the number of patients at risk at each time point.

The median follow-up duration for the entire cohort was 33.1 months. The 1-, 2-, and 3-year DFS rates were 85.9%, 58.7%, and 45.2% in the adjuvant chemotherapy group, compared with 84.6%, 57.1%, and 45.9% in the perioperative chemotherapy group. The median DFS was 28 months in the adjuvant group and 32.5 months in the perioperative group, with no significant difference (HR = 0.99, 95% CI 0.80–1.22; log-rank *P* = 0.945) between the two groups (**Figure 1A**).

Given higher CRS scores indicate the increased postoperative recurrence risk, we performed pre-specified subgroup analyses stratified by CRS score to compare the two regimens. DFS was comparable between the adjuvant and perioperative chemotherapy groups in patients with CRS 3 (HR = 1.02, 95% CI 0.77–1.33; log-rank *P* = 0.881, **Figure 1B**) and CRS 4 (HR = 1.30, 95% CI 0.84–2.01; log-rank *P* = 0.233, **Figure 1C**). In contrast, perioperative chemotherapy significantly prolonged DFS in patients with CRS 5 (HR = 0.54, 95% CI 0.30–0.96; log-rank *P* = 0.039; Bonferroni-corrected *P* = 0.117; **Figure 1D**). After the adjustment for age, gender, T stage, N stage, CEA, and RAS/BRAF status, formal interaction testing indicated that the interaction between perioperative chemotherapy and CRS 5 was significant (HR = 0.49, 95% CI 0.26–0.94; *P* = 0.032), whereas no interaction was observed for CRS 4 (HR = 1.25, 95% CI 0.75–2.09; *P* = 0.399).

**Figure 2:**
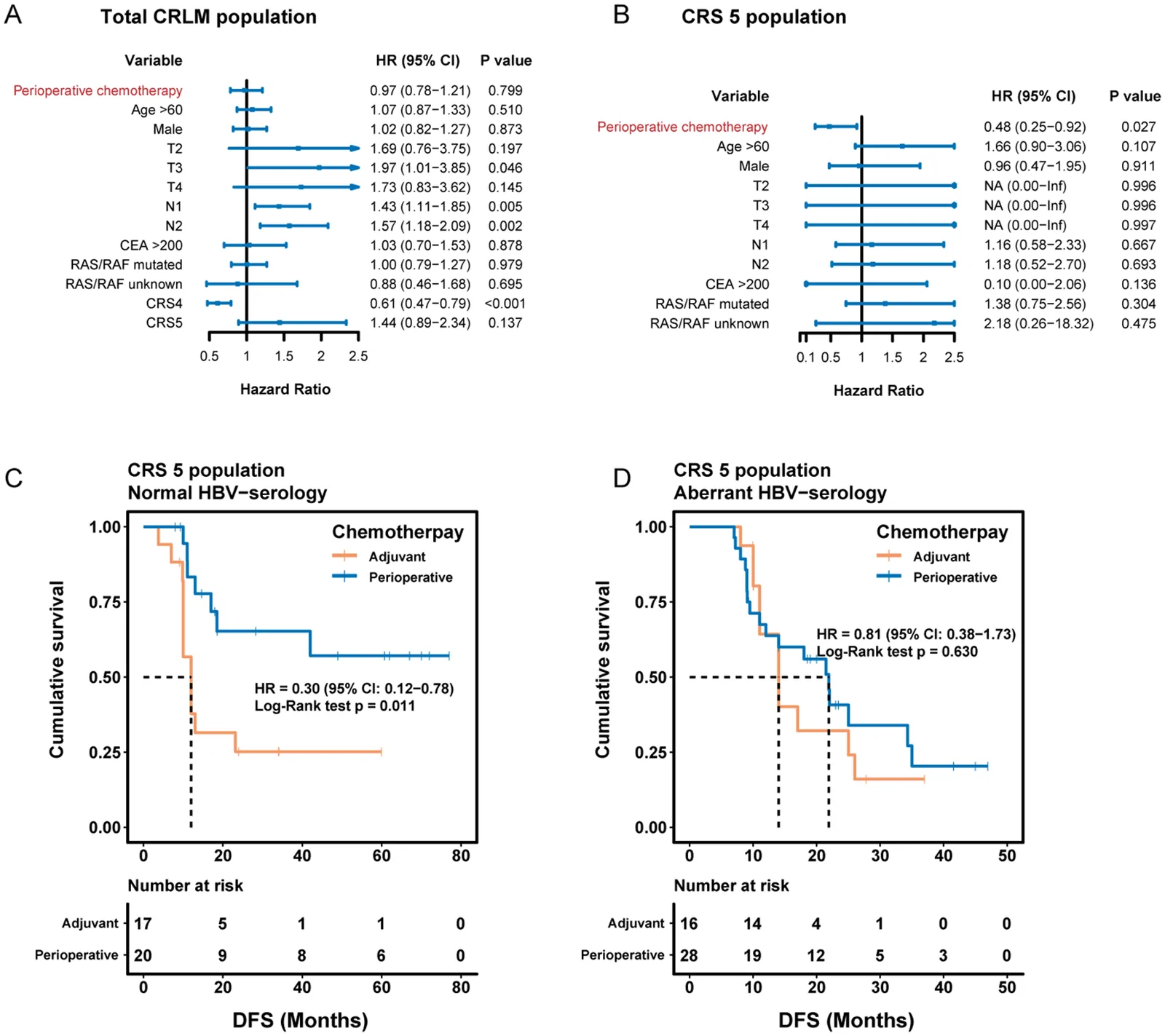
Forest plots and Kaplan-Meier estimates of DFS in CRLM patients. **(A)** Forest plot of adjusted hazard ratios for DFS in the overall cohort. **(B)** Forest plot of adjusted hazard ratios for DFS in the CRS 5 subgroup. **(C)** DFS comparison between perioperative and adjuvant chemotherapy in CRS 5 patients with normal HBV-related serology. **(D)** DFS comparison between perioperative and adjuvant chemotherapy in CRS 5 patients with aberrant HBV-related serology. Note: aberrant HBV-related serology includes CHB and OHB, while normal HBV-related serology means HBV-negative status.

Multivariable Cox regression analyses were presented as forest plots (**Figure 2**). In the overall cohort, perioperative chemotherapy was not independently associated with DFS (adjusted HR = 0.97, 95% CI 0.78–1.21; *P* = 0.799; **Figure 2A**). Likewise, in the CRS 5 subgroup, it remained a significant predictor of improved DFS (adjusted HR = 0.48, 95% CI 0.25–0.92; *P* = 0.027; **Figure 2B**).

### HBV serological status may predict perioperative chemotherapy efficacy in the CRS 5 subgroup

HBV infection can modulate the hepatic microenvironment. Therefore, we aimed to explore its serological status as a potential modifier of the treatment effect within the CRS 5 population. Stratified analysis revealed that in normal HBV-related serology patients with CRS 5, perioperative chemotherapy significantly prolonged DFS compared with adjuvant chemotherapy (HR = 0.30, 95% CI 0.12–0.78; log-rank *P* = 0.014; Bonferroni-corrected *P* = 0.042; **Figure 2C**). In contrast, among aberrant HBV-related serology patients with CRS 5, this survival advantage was abolished (HR = 0.81, 95% CI 0.38–1.73; log-rank *P* = 0.588; **Figure 2D**). Whereas, the interaction between perioperative chemotherapy and HBV infection was of borderline significance (adjusted HR for interaction = 2.93, 95% CI 0.82–10.49; *P* = 0.099).

### HBV infection may attenuate perioperative chemotherapy benefit in CRS 4–5 patients

**Figure 3:**
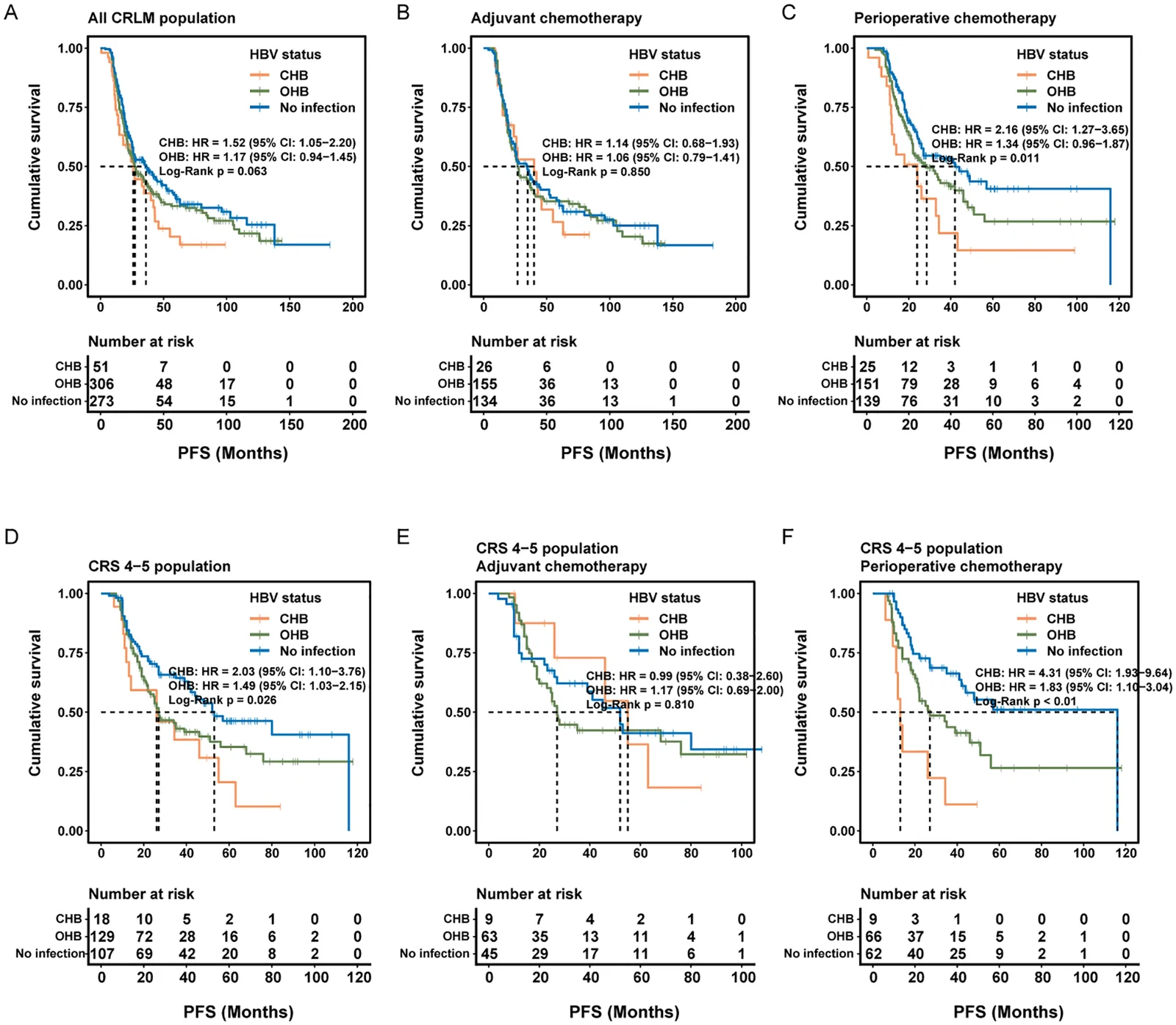
Kaplan-Meier estimates of DFS stratified by HBV infection status and chemotherapy regimen. **(A)** Comparison of DFS between CHB, OHB, and no infected patients in the overall cohort. **(B)** Comparison of DFS between CHB, OHB, and no infected patients in the adjuvant chemotherapy subgroup. **(C)** Comparison of DFS between CHB, OHB, and no infected patients in the perioperative chemotherapy subgroup. **(D)** Comparison of DFS between CHB, OHB, and no infected patients in CRS 4-5 patients. **(E)** Comparison of DFS between CHB, OHB, and no infected patients in CRS 4-5 patients receiving adjuvant chemotherapy. **(F)** Comparison of DFS between CHB, OHB, and no infected patients in CRS 4-5 patients receiving perioperative chemotherapy.

To further characterize the interaction between HBV infection and chemotherapy efficacy, we conduct subgroup analyses stratified by HBV infection status. Based on HBV-related serological profiles, patients were classified into three groups: CHB, OHB, and HBV-negative. Notably, no patient required antiviral therapy due to acute HBV flare-up during the perioperative period. In the overall cohort (**Figure 3A**), CHB showed a trend toward inferior DFS compared with HBV-negative patients (HR = 1.52, 95% CI 1.05–2.20), while OHB did not (HR = 1.17, 95% CI 0.94–1.45). When stratified by chemotherapy regimen, CHB patients also had significantly worse DFS compared with HBV-negative patients (HR = 2.16, 95% CI 1.27–3.65, log-rank *P* = 0.011; Bonferroni-corrected *P* = 0.033; **Figure 3C**). By contrast, in the adjuvant chemotherapy group, HBV status was not associated with DFS (log-rank *P* = 0.850; HR_(CHB)_ = 1.14, 95% CI 0.68–1.93; HR_(OHB)_ = 1.06, 95% CI 0.79–1.41; **Figure 3B**).

We next focused on the high-risk population of CRS 4-5, where the DFS benefit of perioperative chemotherapy was most evident. In this subgroup, DFS differed significantly according to HBV status. Both CHB and OHB patients had worse DFS than HBV-negative patients (log-rank *P* = 0.026; Bonferroni-corrected *P* = 0.078; HR_(CHB)_ = 2.03, 95% CI 1.10–3.76; HR_(OHB)_ = 1.49, 95% CI 1.03–2.15; **Figure 3D**). When stratified by chemotherapy, the association was stronger in the perioperative group (log-rank *P* = 0.00055; Bonferroni-corrected *P* = 0.0017; HR_(CHB)_ = 4.31, 95% CI 1.93–9.64; HR_(OHB)_ = 1.83, 95% CI 1.10–3.04; **Figure 3F**). Similarly, no significant difference in DFS was observed among CHB, OHB, and HBV-negative patients in the adjuvant chemotherapy group (log-rank *P* = 0.810; HR_(CHB)_ = 0.99, 95% CI 0.38–2.60; HR_(OHB)_ = 1.17, 95% CI 0.69–2.00; **Figure 3E**). Formal interaction testing in the CRS 4-5 population confirmed a significant interaction between perioperative chemotherapy and CHB status (adjusted HR for interaction = 4.65, 95% CI 1.25–17.34; *P* = 0.022), but not for OHB (HR = 1.45, 95% CI 0.67–3.11; *P* = 0.346). Finally, in the CRS 3 subgroup, HBV status was not associated with DFS in either the perioperative chemotherapy group (**Supplementary Figure 2A**) or the adjuvant chemotherapy group (**Supplementary Figure 2B**).

### Impact of HBV infection on overall survival in CRLM patients

While multiple clinical trials have demonstrated that chemotherapy improves DFS but not OS in patients with CRLM (21–23), we nevertheless evaluated whether HBV infection influenced OS outcomes in our cohort.

In the overall cohort, the log-rank test did not show a significant difference in OS among CHB, OHB, and HBV-negative patients (*P* = 0.130). However, the HR for CHB was 1.52 (95% CI 1.05–2.20) which suggested a potential association with inferior OS (**Supplementary Figure 1A**). When stratified by chemotherapy regimen, the detrimental effect of CHB on OS was more pronounced in the perioperative chemotherapy group (log-rank *P* = 0.140; HR_(CHB)_ = 2.16, 95% CI 1.27–3.65; **Supplementary Figure 1C**). In the CRS 4-5 population, CHB was consistently associated with worse OS, both in the overall CRS 4-5 cohort (log-rank *P* = 0.055; Bonferroni-corrected *P* = 0.165; HR_(CHB)_ = 2.03, 95% CI 1.10–3.76; **Supplementary Figure 1D**) and in those receiving perioperative chemotherapy (log-rank P = 0.002; Bonferroni-corrected *P* = 0.006; HR_(CHB)_ = 4.22, 95% CI 1.76–10.09; **Supplementary Figure 1F**). In contrast, among patients receiving adjuvant chemotherapy, HBV status was not significantly associated with OS (**Supplementary Figure 1B**, **Supplementary Figure 1E**). Similarly, in the CRS 3 subgroup, no association was observed between HBV status and OS (**Supplementary Figure 2C**, **Supplementary Figure 2D**). Collectively, the inferior prognostic outcomes of CHB may suggest an underlying mechanism of chemoresistance.

### CRLM with HBV infection enriches TSPAN8^+^ stem-like epithelium

**Figure 4:**
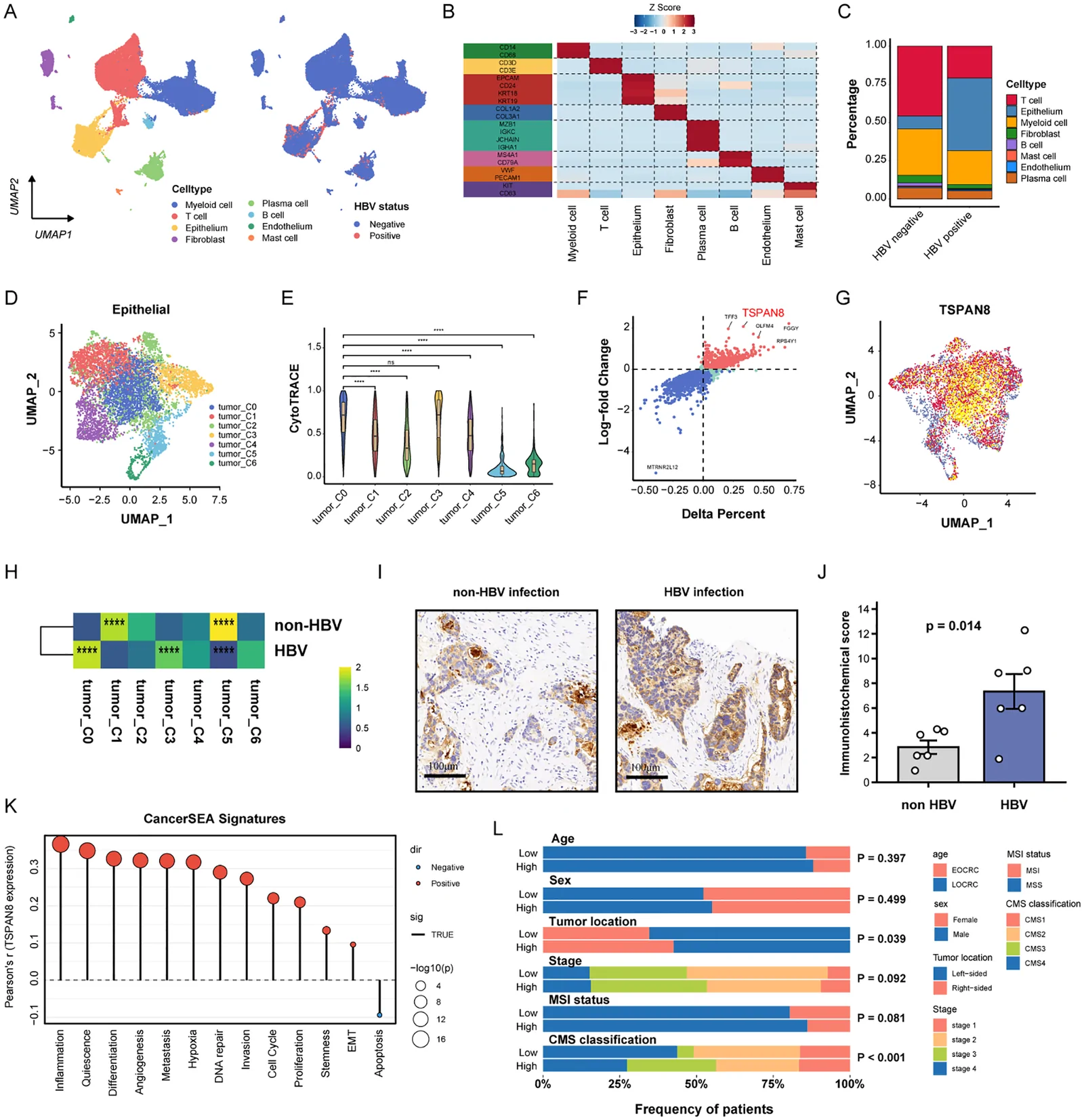
TSPAN8^+^ stem-like epithelial subpopulation was enriched in HBV infected sample. **(A)** UMAP visualization of eight cell types annotated in HBV-negative and HBV-positive CRLM samples. **(B)** The expression of canonical marker genes across the eight annotated cell types. **(C)** The proportional composition of each cell type between the two groups. **(D)** UMAP visualization of six distinct epithelial subclusters. **(E)** CytoTRACE2 analysis revealing the developmental potential of each epithelial subcluster. **(F)** Differential expression analysis identifying top upregulated genes in HBV-positive epithelial cells. **(G)** The expression distribution of TSPAN8 across epithelial subclusters. **(H)** Ro/e analysis demonstrating the enriched epithelial subcluster in HBV-positive samples. **(I)** Representative immunohistochemical images of TSPAN8 staining in CRLM tissues. **(J)** Quantification of TSPAN8 staining scores in the two groups. **(K)** Correlation between TSPAN8 expression and functional states. **(L)** The clinical features of patients with high TSPAN8 expression.

Thereafter, single-cell RNA sequencing was conducted to explore the potential mechanism underlying the blunted benefit of perioperative chemotherapy in HBV-infected CRLM patients. A total of nine CRLM samples were analyzed, including two HBsAg-positive samples and seven HBsAg-negative samples. By classic cell type markers, we annotated 8 major cell populations (**Figures 4A, B**). Cell composition analysis demonstrated that HBV-positive CRLM samples contained higher proportion of epithelial cells (**Figure 4C**). Thus, we focused on the heterogeneity of tumor epithelium. Epithelial cells were further divided into 6 distinct subpopulations (**Figure 4D**). CytoTRACE2 analysis was used to infer the developmental potential of these subpopulations, and revealed that the C0 cluster was most undifferentiated (**Figure 4E**). Differential expression analysis identified TSPAN8, TFF3, RPS4Y1, and FGGY as the top upregulated genes in HBV-positive epithelium (**Figure 4F**), and TSPAN8 was predominantly expressed in the C0 cluster (**Figure 4G**). Notably, Ro/e analysis indicated that the C0 cluster was significantly enriched in HBV-positive samples (**Figure 4H**). To validate, we performed immunohistochemical staining on 12 CRLM metastatic samples. It indicated that TSPAN8 was predominantly localized in tumor epithelial cells. And both the staining intensity and the proportion of positive cells were significantly increased in HBV-infected samples (**Figures 4I, J**).

TSPAN8 was positively correlated with stemness and quiescence scores, whereas a negative correlation was observed with apoptosis. Besides, the aggressive tumor traits were also positively associated with TSPAN8 expression, including angiogenesis, metastasis, invasion, and hypoxia (**Figure 4K**). To validate, we evaluated TSPAN8 correlation with 26 independent stemness-related gene sets, and TSPAN8 showed significant positive correlations with the vast majority of these signatures (**Supplementary Figure 3**). Finally, TSPAN8 high-expression tumors were more frequently located in the right-sided colon and were preferentially enriched in the Consensus Molecular Subtype 3 classification (**Figure 4L**).

### TSPAN8 promotes the drug resistance to 5-fluorouracil in CRLM

**Figure 5:**
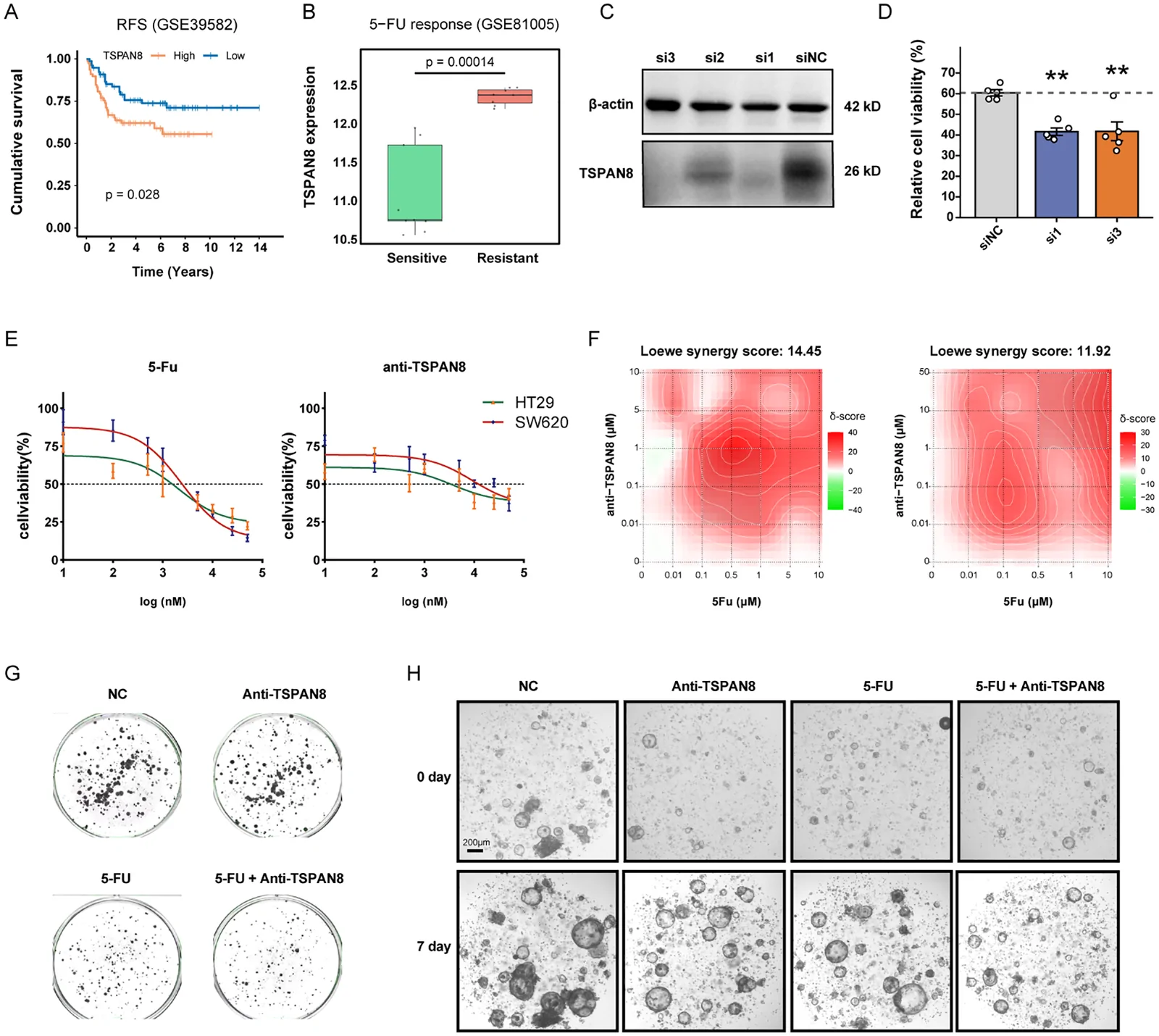
TSPAN8 promotes resistance to 5-fluorouracil in CRLM. **(A)** The prognostic impact of TSPAN8 expression in patients receiving chemotherapy. **(B)** The change of TSPAN8 mRNA expression in HCT-8 cells with acquired 5-FU resistance. HCT-8 cells with acquired 5-fluorouracil resistance were derived from the GSE81005 dataset. Parental HCT-8 and HCT-8/5-FU cells were cultured. RNA was extracted and gene expression was profiled using Affymetrix microarrays (GPL15207). **(C)** Western blot validation of TSPAN8 knockdown in SW620 cells. **(D)** The relative cell viability of TSPAN8-silenced SW620 after 5-FU treatment. **(E)** Dose-response curves of 5-fluorouracil and anti-TSPAN8 antibody in HT29 and SW620 cells. **(F)** The synergistic effects between 5-fluorouracil and anti-TSPAN8 antibody in HT29 and SW620 cells. **(G)** Colony formation assay of SW620 cells treated with 5-fluorouracil, anti-TSPAN8 antibody, or their combination. **(H)** Bright-field images of patient-derived CRLM organoids after 7-day treatment with 5-fluorouracil, anti-TSPAN8 antibody, or combination therapy.

Previous studies had reported TSPAN8-mediated stemness is implicated in chemoresistance (24–26). In GSE39582 cohort, patients with low TSPAN8 expression had a markedly improved recurrence-free survival when chemotherapy was administered (**Figure 5A**). Besides, TSPAN8 expression was significantly elevated in HCT-8 cells with acquired 5-fluorouracil resistance (**Figure 5B**).

Accordingly, we sought to investigate the effect of TSPAN8 on 5-fluorouracil, a first-line chemotherapeutic agent commonly used in colorectal cancer. We then silenced TSPAN8 in SW620 cell line of colorectal cancer by siRNA-mediated knockdown (**Figure 5C**). After 5-fluorouracil treatment, TSPAN8-silenced SW620 exhibited significantly lower relative cell viability compared with control siRNA-transfected cells (**Figure 5D**). As TSPAN8 belongs to a family of proteins with four transmembrane domains, it is a promising therapeutic target for monoclonal antibody therapy (27). We therefore evaluated the dose-response curves of 5-fluorouracil and anti-TSPAN8 antibody in HT29 and SW620 cells (**Figure 5E**). Synergy between the two agents was assessed by SynergyFinder (https://synergyfinder.aittokallio.group/), and the Loewe synergy scores exceeded 10 in HT29 and SW620 cells. Thus, it indicates strong synergistic effects between 5-fluorouracil and anti-TSPAN8 antibody (**Figure 5F**). And we also found that combined 5-fluorouracil and anti-TSPAN8 antibody treatment markedly reduced the colony formation of SW620 (**Figure 5G**). Moreover, in a patient-derived CRLM organoid model, 7-day combination treatment significantly decreased organoid size and count compared with control and monotherapy groups (**Figure 5H**).

### Crosstalk between TSPAN8^+^ epithelium and SPP1^+^ macrophage was identified in HBV infected CRLM

**Figure 6:**
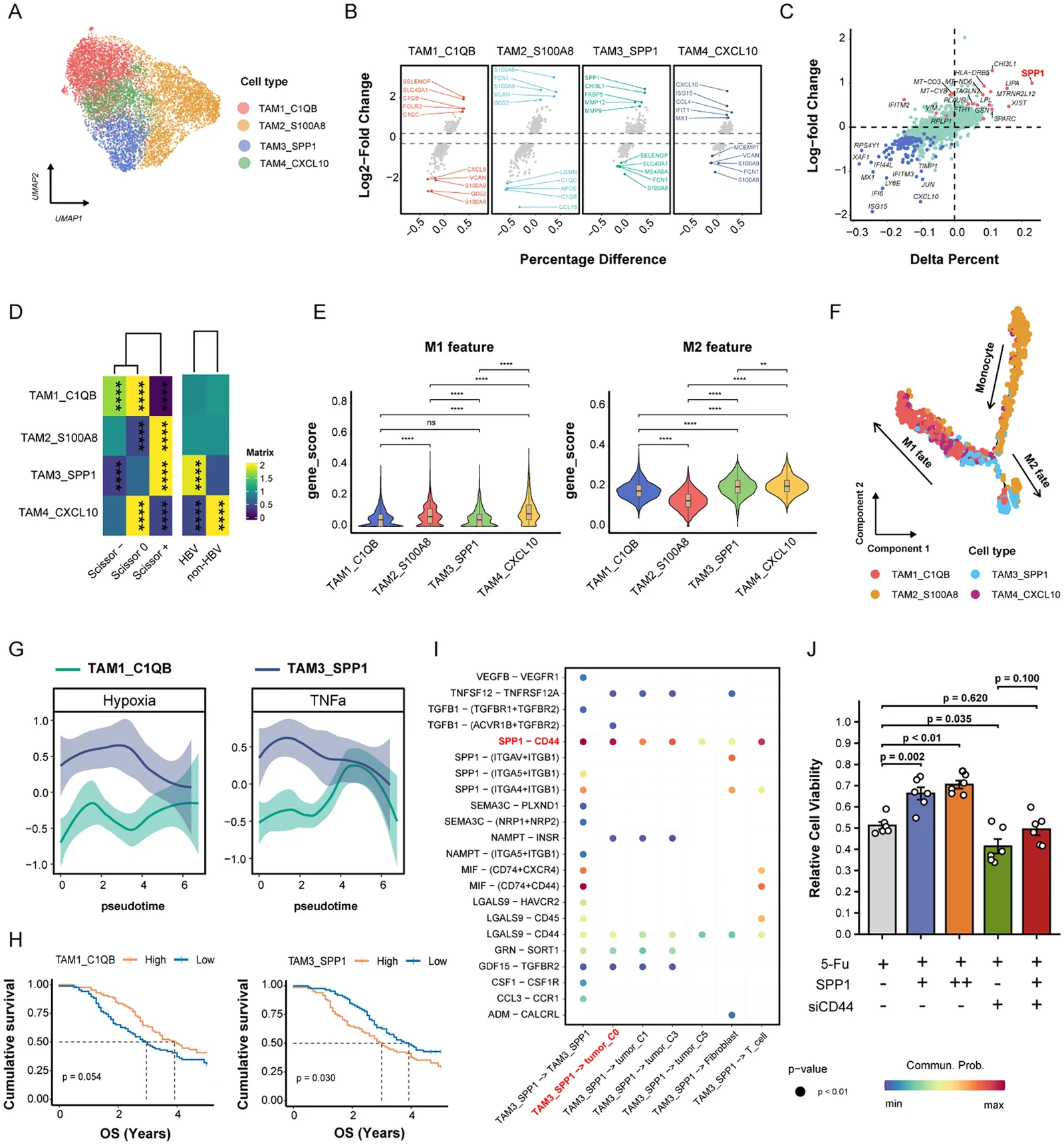
Crosstalk between TSPAN8^+^ epithelium and SPP1^+^ macrophage in HBV-infected CRLM. **(A)** UMAP visualization of 4 distinct tumor-associated macrophage (TAM) subcluster. **(B)** Heatmap of top 5 gene expression across the four TAM subclusters. **(C)** Differentially expressed genes between HBV-positive and HBV-negative TAM. **(D)** Enrichment of TAM3_SPP1 in HBV-positive samples and its association with overall survival by Ro/e analysis. **(E)** M1 and M2 polarization scores across TAM subclusters. **(F)** Pseudotime trajectory analysis of TAM differentiation. **(G)** Hypoxia and TNFα pathway activities along pseudotime. **(H)** Kaplan-Meier curves of overall survival in patients receiving chemotherapy. **(I)** Ligand-receptor interactions between tumor epithelial cells and TAM3_SPP1. **(J)** Cell viability of SW620 cells exposed to 5-fluorouracil (5-FU). Cells were treated with recombinant SPP1 protein alone (2 μg/mL, and 5 μg/mL, respectively), CD44 knockdown, and CD44 knockdown plus recombinant SPP1 protein. After 48 hours of treatment, cell viability was measured by CCK-8 assay.

To characterize the macrophage in CRLM, we analyzed 6,351 myeloid cells and resolved them into four distinct tumor-associated macrophage (TAM) subclusters (**Figures 6A, B**). Differential expression analysis revealed that SPP1 was significantly upregulated in HBV-positive samples (**Figure 6C**). In addition, TAM3_SPP1 was markedly enriched in HBV-positive liver metastases, and was associated with poorer OS by Ro/e method (**Figure 6D**). TAM3_SPP1 exhibited a significantly elevated M2 polarization score and a reduced M1 score (**Figure 6E**). Conversely, TAM2_S100A8 showed the opposite pattern with high M1 and low M2 scores. As S100A8 is known as monocyte-associated marker, thus TAM2_S100A8 was considered the root of macrophage differentiation, and two distinct differentiation branches were identified (**Figure 6F**).

The branch where TAM1_C1QB and TAM4_CXCL10 were mainly concentrated was identified as the trajectory toward M1 polarization, while TAM3_SPP1 was concentrated in the trajectory toward M2 polarization (**Figure 6F**). With the increase of pseudotime, the hypoxia and TNFα pathways in TAM3_SPP1 were higher than those in TAM1_C1QB (**Figure 6G**). And the infiltration of TAM3_SPP1 also affected the response of CRLM patients to chemotherapy. In the GSE159216 cohort, the infiltration of TAM3_SPP1 was associated with poorer OS in patients receiving chemotherapy (**Figure 6H**). In contrast, the infiltration level of TAM1_C1QB was associated with better OS with marginal significance (**Figure 6H**).

Cell–cell communication analysis revealed robust ligand–receptor interactions between tumor epithelial cells (C0 cluster) and TAM3_SPP1, and found the SPP1–CD44 axis was the most prominent interaction (**Figure 6I**). It indicated that TSPAN8^+^ cells may originate from the interaction between CD44-positive carcinoma cells and SPP1-positive TAMs. To validate this interaction, SW620 cells were treated with recombinant SPP1 protein. SPP1 significantly increased cell viability under 5-fluorouracil exposure (**Figure 6J**). CD44 knockdown alone significantly reduced cell viability under 5-fluorouracil exposure, while the addition of SPP1 and failed to restore cell viability (**Figure 6J**). These results indicate that SPP1-CD44 signaling mediates chemoresistance in CRLM.

## Discussion

Perioperative chemotherapy is widely considered the optimal treatment for patients with resectable CRLM. However, in real-world clinical practice, few CRLM patients receive perioperative chemotherapy mainly due to concerns about severe liver comorbidities. And no robust evidence shows survival benefit of perioperative chemotherapy for all high-risk patients (8, 9). In the present study, we found no significant advantage in relapse risk between perioperative chemotherapy and adjuvant chemotherapy in patients with resectable CRLM. The result highlighted the real-world challenge of formulating personalized chemotherapy strategies for CRLM.

CRS is a classic clinical system that reflects the recurrence risk and serves as an indicator for chemotherapy selection in CRLM. Based on preplanned subgroup analyses stratified by CRS, we compared the effects of adjuvant and perioperative chemotherapy in CRLM patients. For the first time, we observed that perioperative chemotherapy significantly improved DFS only in the CRS 5 subgroup (25 vs. 13 months, *P* = 0.039) compared with adjuvant chemotherapy.

Interestingly, the survival benefit of perioperative chemotherapy in high-risk patients are highly associated with the HBV-related serological profiles. Although HBV infection is well recognized as a stratification factor in hepatocellular carcinoma, it is often overlooked in metastatic liver tumors. In the CRS 4–5 population, a significant interaction between perioperative chemotherapy and CHB status was observed (adjusted HR for interaction = 4.65, 95% CI 1.25–17.34; *P* = 0.022) by formal interaction testing. Thus, the existence of HBV infection may serve as a clinically relevant modifier of perioperative chemotherapy efficacy in CRS 4–5 CRLM patients, and warrants consideration in treatment decision-making.

Previous transcriptomics have depicted the complex TME of colorectal cancer, yet the TME of HBV-positive CRLM remains poorly understood. Recent evidence from hepatocellular carcinoma has demonstrated that HBV-infected tumors exhibit a significant enrichment of SPP1-high macrophages (28). Consistent with this observation, our study is the first to identify that SPP1^+^ TAMs are enriched in HBV-positive metastatic liver lesions. TAM have well-documented heterogeneous functions in tumor progression (29–31), and SPP1^+^ TAM are suspected to play roles in angiogenesis and matrix remodeling in multiple tumors (32, 33). Our findings further demonstrate that SPP1^+^ TAMs are not only a major component of the liver metastatic TME, but also contribute to the maintenance of chemoresistance through the SPP1-CD44 axis.

Moreover, we observed a consistent positive correlation between TSPAN8 expression and stemness across multiple independent gene set-based stemness scores. And the colocalization with the stemness marker CD44 is consistent with previous reports, which a binding site within the Tspan8 promoter can be crosslinked with CD44 to promote Tspan8 mRNA transcription (34). The highly stem-like epithelial phenotype observed in CRLM may thus represent a crucial mechanism underlying HBV infection-associated chemoresistance in CRLM (35). Functionally, TSPAN8 knockdown significantly reduced chemoresistance of colorectal cancer cells, and anti-TSPAN8 antibody treatment exhibited strong synergistic effects with 5-fluorouracil.

Our findings lay a valuable foundation for improving the understanding of crosstalk between cancer cells and SPP1^+^ TAM, and for developing novel therapeutic strategies for CRLM. Collectively, it enhances the understanding of how HBV infection modulates the tumor microenvironment, and identifies TSPAN8 as a potential therapeutic target.

The study has several limitations. First, its retrospective nature limits the ability to draw causal inferences. Second, our study only enrolled high-risk (CRS ≥3) CRLM patients, and 630 patients were included in the analysis after PSM. The cohort is one of the largest CRLM series focusing on high-risk patients. However, due to the relatively low prevalence of CRS 5 in CRLM, this population still accounted for only 10.5% to 15.2% in our cohort. The limited sample size of CRS 5 patients may restrict the statistical power and generalizability of our subgroup findings. Finally, clinically significant liver events (viral load, antiviral therapy, and hepatic fibrosis) were too few in our cohort to allow meaningful subgroup analyses. The optimal use of antiviral or hepatoprotective therapy in this setting warrants further investigation.

## Conclusion

The study identifies the CRS score as a useful tool to distinguish patients who may benefit from perioperative chemotherapy. The HBV-related serology may serve as a crucial indicator of worse prognosis in patients receiving this regimen. Although the mechanistic exploration is suggestive, it remains exploratory and warrant validation in prospective studies.

## Data Availability

The original contributions presented in the study are included in the article/**Supplementary Material**. Further inquiries can be directed to the corresponding author.

## Supplementary material

Supplementary Figure 1 Kaplan-Meier estimates of overall survival (OS) stratified by HBV infection status and chemotherapy regimen. **(A)** Comparison of OS between CHB, OHB, and no infected patients in the overall cohort. **(B)** Comparison of OS between CHB, OHB, and no infected patients in the adjuvant chemotherapy subgroup. **(C)** Comparison of OS between CHB, OHB, and no infected patients in the perioperative chemotherapy subgroup. **(D)** Comparison of OS between CHB, OHB, and no infected patients in CRS 4-5 patients receiving adjuvant chemotherapy. **(E)** Comparison of OS between CHB, OHB, and no infected patients in CRS 4-5 patients receiving perioperative chemotherapy.

Supplementary Figure 2 Impact of HBV infection in CRLM patients with CRS 3. **(A)** Comparison of DFS between CHB, OHB, and no infected patients in CRS 3 patients receiving adjuvant chemotherapy. **(B)** Comparison of DFS between CHB, OHB, and no infected patients in CRS 3 patients receiving perioperative chemotherapy. **(C)** Comparison of OS between CHB, OHB, and no infected patients in CRS 3 patients receiving adjuvant chemotherapy. **(D)** Comparison of OS between CHB, OHB, and no infected patients in CRS 3 patients receiving perioperative chemotherapy.

Supplementary Figure 3 Correlation between TSPAN8 and 26 stemness gene set score.

Supplementary Table 1 Baseline characteristics of the study population before propensity score matching.
