# Supplementary material for: Chronic hepatitis B and perioperative chemotherapy efficacy in colorectal liver metastases: an exploratory analysis of clinical and mechanistic evidence
Source: Front Immunol. 2026 Sep 4;17:1914602. doi: 10.3389/fimmu.2026.1914602 (PMC13585926; doi:10.3389/fimmu.2026.1914602)
Supplement: Supplementary file 1 [file Table1.docx]

**Supplemental Table 1.** Baseline characteristics of the study population before propensity score matching.

| **Variables** | **Category** | **Adjuvant chemotherapy**  **(N = 398)** | **Perioperative chemotherapy (N = 424)** | **SMD^*^** | ***P* value** |
| --- | --- | --- | --- | --- | --- |
| **Gender** | Male | 273 (68.6%) | 275 (64.9%) | 0.079 | 0.256 |
|  | Female | 125 (31.4%) | 149 (35.1%) |  |  |
| **Age** | **≥** 60 years | 195 (49.0%) | 171 (40.3%) | 0.175 | 0.012 |
|  | **<** 60 years | 203 (51.0%) | 253 (59.7%) |  |  |
| **Histology** | Adenocarcinoma | 383 (96.0%) | 319 (98.3%) | 0.139 | 0.067 |
|  | mucinous Adenocarcinoma | 15 (4.0%) | 5 (1.7%) |  |  |
| **pT** | T1 | 34 (8.5%) | 25 (5.9%) | 0.217 | < 0.001 |
|  | T2 | 52 (13.1%) | 29 (6.8%) |  |  |
|  | T3 | 248 (62.3%) | 265 (62.5%) |  |  |
|  | T4 | 64 (16.1%) | 105 (24.8%) |  |  |
| **pN** | N0 | 159 (39.9%) | 172 (40.6%) | 0.040 | 0.835 |
|  | N1 | 133 (33.4%) | 148 (34.9%) |  |  |
|  | N2 | 106 (26.6%) | 104 (24.5%) |  |  |
| **Location** | Right | 275 (69.1%) | 267 (63.0%) | 0.130 | 0.117 |
|  | Left | 117 (29.4%) | 145 (34.2%) |  |  |
|  | Rectum | 6 (1.5%) | 12 (2.8%) |  |  |
| **Tumor size** | **≥** 3 cm | 264 (66.3%) | 278 (65.6%) | 0.016 | 0.817 |
|  | **<** 3 cm | 134 (33.7%) | 146 (34.4%) |  |  |
| **Metastasis size** | Mean ± SD | 4.11 ± 1.87 cm | 3.97 ± 2.13 cm | 0.069 | 0.318 |
| **Metastasis number** | **≥** 2 | 318 (79.9%) | 385 (90.8%) | 0.312 | < 0.001 |
|  | **< 2** | 80 (20.1%) | 39 (9.2%) |  |  |
| **CRS score** | 3 | 209 (52.5%) | 209 (52.5%) | 0.106 | 0.298 |
|  | 4 | 94 (23.6%) | 122 (28.8%) |  |  |
|  | 5 | 95 (23.9%) | 101 (23.8%) |  |  |
| **RAS/BRAF status** | Mutation | 114 (28.6%) | 139 (32.8%) | 0.090 | 0.199 |
|  | Wild-type | 284 (71.4%) | 285 (67.2%) |  |  |
| **HBV status** | CHB | 41 (10.3%) | 31 (7.3%) | 0.106 | 0.199 |
|  | OHB | 189 (47.5%) | 222 (52.4%) |  |  |
|  | Negative | 168 (42.2%) | 171 (40.3%) |  |  |
| **Liver function** | Abnormal | 8 (2%) | 4 (0.9%) | 0.089 | 0.199 |
|  | Normal | 390 (98%) | 422 (99.1%) |  |  |

Notes:

SMD*: standardized mean difference. For multi‑level categorical variables, the largest SMD among all levels is reported. SMD < 0.1 indicates good balance, and SMD < 0.2 is generally considered acceptable.
